# 西罗莫司成功治疗复发难治性特发性多中心型Castleman病1例

**DOI:** 10.3760/cma.j.cn121090-20240119-00034

**Published:** 2024-05

**Authors:** 雨菡 高, 路 张, 剑 李

**Affiliations:** 中国医学科学院，北京协和医学院，北京协和医院血液内科，北京 100730 Department of Hematology, Peking Union Medical College Hospital, Chinese Academy of Medical Sciences and Peking Union Medical College, Beijing 100730, China

患者，男，35岁，因“多发淋巴结肿大2个月，发热1个月”于2017年7月就诊于我院。血常规：WBC 8.98×10^9^/L、HGB 110 g/L、PLT 72×10^9^/L；平均红细胞体积71.7 fl、网织红细胞比例2.05％；铁蛋白384 ng/ml、总铁结合力1300 µg/L、转铁蛋白饱和度10.8％。总蛋白45 g/L，白蛋白23 g/L，碱性磷酸酶930 U/L，肌酐160 µmol/L，C反应蛋白（CRP）258.99 mg/L，血沉78 mm，白细胞介素-6（IL-6）65.1 ng/L。免疫球蛋白：IgG 6.74 g/L、IgA 1.00 g/L、IgM 0.40 g/L。血清免疫固定电泳阴性。抗核抗体谱阴性。HIV抗体及人类疱疹病毒8（HHV-8）抗体阴性，EB病毒DNA检测阴性。颈部右侧淋巴结活检病理：Castleman病（混合型）；免疫组化：CD20（+），CD21（+），bcl-2（+），CD38（局灶+），CD138（局灶+），Ki-67（增殖指数20％）。查体：贫血貌；颈部、颏下、颌下、锁骨上、腹股沟多发淋巴结肿大，质软，界清；腹部膨隆，移动性浊音阴性；肝肋缘下5 cm，脾肋缘下10 cm。CT示双侧腋窝、肺门及纵隔多发肿大淋巴结，腹膜后及肠系膜周多发肿大淋巴结，肝大，脾大，腹腔少量积液。明确诊断特发性多中心型Castleman病-非特指型（iMCD-NOS）。2017年8月起予TCP（沙利度胺+环磷酰胺+泼尼松）方案治疗7个疗程，每4周1个疗程。治疗期间患者体温逐渐降至正常，但生化指标改善欠佳（CRP 183.83 mg/L、HGB 108 g/L、白蛋白23 g/L）。CT示横膈上下多发淋巴结肿大，部分较前略缩小；根据国际Castleman病协作网络（CDCN）疗效评估标准，评估为疾病稳定（SD）。2018年5月起更换为BCD（硼替佐米+环磷酰胺+地塞米松）方案治疗9个疗程，每4周1个疗程。评估患者整体疗效为完全缓解（CR）。2023年1月患者再次出现乏力、发热，浅表淋巴结肿大，CRP 257.32 mg/L、HGB 115 g/L、白蛋白43 g/L、肌酐57 µmol/L，考虑疾病进展。2023年3月起调整治疗方案为西罗莫司1 mg每日1次联合地塞米松20 mg每周1次。用药后第2个月查西罗莫司谷浓度4.5 µg/L，调整西罗莫司为1.5 mg每日1次，复查西罗莫司谷浓度5.9 µg/L，后续维持西罗莫司为1.5 mg每日1次剂量。用药第3个月起将地塞米松减至10 mg每周1次，第4个月减至5 mg每周1次。自第5个月起停用地塞米松，予西罗莫司1.5 mg每日1次单药治疗，维持至今。期间检测西罗莫司谷浓度5～15 µg/L。目前患者症状完全缓解，生化指标：CRP 7.27 mg/L，HGB 145 g/L，白蛋白49 g/L，肌酐63 µmol/L。CT未发现肿大淋巴结。考虑治疗有效，整体评估为CR，患者仍继续接受西罗莫司单药治疗。

讨论：iMCD是一类具有特征性病理改变的罕见淋巴增殖性疾病，以IL-6在内的细胞因子风暴驱动的多克隆性淋巴细胞增殖、多区域淋巴结肿大、全身性炎症和多器官功能障碍是该病的主要特征。CDCN指南推荐IL-6靶向治疗作为iMCD的一线治疗选择。基于药物可及性，本例患者采用了TCP、BCD方案作为前线治疗方案，疾病进展后更换为西罗莫司治疗，患者症状得到迅速改善，没有出现肾功能损伤等不良反应，仅使用西罗莫司单药口服即可维持持续缓解状态。

